# The β-d-Endoglucuronidase Heparanase Is a Danger Molecule That Drives Systemic Inflammation and Correlates with Clinical Course after Open and Endovascular Thoracoabdominal Aortic Aneurysm Repair: Lessons Learnt from Mice and Men

**DOI:** 10.3389/fimmu.2017.00681

**Published:** 2017-06-12

**Authors:** Lukas Martin, Alexander Gombert, Jianmin Chen, Julia Liebens, Julia Verleger, Johannes Kalder, Gernot Marx, Michael Jacobs, Christoph Thiemermann, Tobias Schuerholz

**Affiliations:** ^1^Department of Intensive Care and Intermediate Care, RWTH University Hospital Aachen, Aachen, Germany; ^2^The William Harvey Research Institute, Barts and The London School of Medicine and Dentistry, Queen Mary University of London, London, United Kingdom; ^3^Department of Vascular Surgery, European Vascular Center Aachen-Maastricht, RWTH University Hospital Aachen, Aachen, Germany; ^4^Department of Anesthesia and Intensive Care, University Hospital Rostock, Rostock, Germany

**Keywords:** glycosaminoglycan, vascular surgery, syndecan-1, heparan sulfate, heparanase, perioperative care

## Abstract

Thoracoabdominal aortic aneurysm (TAAA) is a highly lethal disorder requiring open or endovascular TAAA repair, both of which are rare, but extensive and complex surgical procedures associated with a significant systemic inflammatory response and high post-operative morbidity and mortality. Heparanase is a β-d-endoglucuronidase that remodels the endothelial glycocalyx by degrading heparan sulfate in many diseases/conditions associated with systemic inflammation including sepsis, trauma, and major surgery. We hypothesized that (a) perioperative serum levels of heparanase and heparan sulfate are associated with the clinical course after open or endovascular TAAA repair and (b) induce a systemic inflammatory response and renal injury/dysfunction in mice. Using a reverse-translational approach, we assessed (a) the serum levels of heparanase, heparan sulfate, and the heparan sulfate proteoglycan syndecan-1 preoperatively as well as 6 and 72 h after intensive care unit (ICU) admission in patients undergoing open or endovascular TAAA repair and (b) laboratory and clinical parameters and 90-day survival, and (c) the systemic inflammatory response and renal injury/dysfunction induced by heparanase and heparan sulfate in mice. When compared to preoperative values, the serum levels of heparanase, heparan sulfate, and syndecan-1 significantly transiently increased within 6 h of ICU admission and returned to normal within 72 h after ICU admission. The kinetics of any observed changes in heparanase, heparan sulfate, or syndecan-1 levels, however, did not differ between open and endovascular TAAA-repair. Postoperative heparanase levels positively correlated with noradrenalin dose at 12 h after ICU admission and showed a high predictive value of vasopressor requirements within the first 24 h. Postoperative heparan sulfate showed a strong positive correlation with interleukin-6 levels day 0, 1, and 2 post-ICU admission and a strong negative correlation with lactate clearance during the first 6 h post-ICU admission. Moreover, systemic administration of heparanase and heparan sulfate induced an inflammatory response and a small degree of renal dysfunction in mice. In conclusion, these results suggest that heparanase and heparan sulfate exhibit a substantial role as clinically relevant danger molecules and may serve as both, promising biomarkers and therapeutic targets in patients undergoing open or endovascular TAAA repair and, indeed, other conditions associated with significant systemic inflammation.

## Introduction

Thoracoabdominal aortic aneurysm (TAAA) is a life-threatening condition with an incidence of about 10 new thoracoabdominal aneurysms per 100,000 person-years (1). The 5-year survival of untreated patients is 10–20%, due to a high proportion of fatal rupture (2). The introduction of cardiopulmonary bypass (CPB) in the early 1960s enabled ongoing organ perfusion and oxygenation during extensive and time-consuming operations of the heart and/or aorta and formed the basis for open surgical TAAA repair, which remains the preferred treatment of choice for TAAA in many institutions worldwide. Although high volume centers have achieved operative mortality rates of less than 10%, the perioperative morbidity remains high (3, 4). Injury/dysfunction of lung, liver, and kidney requiring temporary or permanent hemodialysis are the most frequently perioperative complications (4). Several, mostly CPB-related factors, such as ischemia-reperfusion (I/R) injury, hemodilution, and intravascular hemolysis, all of which result in overwhelming systemic inflammatory response, have been extensively studied to gain a better understanding of their specific roles(s) in the development of these postoperative complications (5). The underlying pathophysiological factors/mechanism, however, remain unclear. During the last 10 years, endovascular techniques using fenestrated and branched stent grafts have been established. Endovascular treatment is achieved by transluminal placement of one or more prostheses across the longitudinal extent of the lesion, which bridges the aneurysmal sac (6). Although, at a first glance, the injury associated with this technique seems to be less compared to the convectional open surgical approach, a significant postoperative inflammatory response, however, leading to the so called postimplantation syndrome (PIS), can bee seen in about one-third of patients undergoing endovascular TAAA repair. PIS leads to the development of serious complication similar to those seen after open surgical TAAA repair (injury/dysfunction of lung, liver, and kidney) (7–9). Again, the underlying pathophysiological factors/mechanism, however, remain unclear.

As a result of its unique position directly between the blood and the vessel wall, the endothelial glycocalyx plays a pivotal role during inflammatory processes, both as active participant and victim (10). This fragile endothelial surface layer has a thickness from 1 to 3 µm and consists of glycosaminoglycans with a core membrane-bound protein and attached heparan sulfate side chains (11). During inflammation caused by trauma/injury, the glycocalyx is “activated” and becomes both target and propagator of the systemic inflammatory response (12). Here, the degradation of the fragile glycocalyx by sheddases plays a key role (10). The mammalian β-d-endoglucuronidase heparanase, cloned in 1999 (13), represents one of these enzymes. Mammalian cells (i.e., endothelial cells, leukocytes, and platelets) express primarily a single dominant functional heparanase enzyme (heparanase-1) (14). In contrast to a second cloned and sequenced heparanase (heparanase-2) (15), heparanase-1 is a multitasking enzyme exhibiting enzymatic and non-enzymatic activities (14). For simplification, throughout this manuscript, we will refer the β-d-endoglucuronidase heparanase-1 as heparanase. Initially synthesized as an inactive 65-kDa precursor, heparanase becomes processed into its active 50-kDa form by lysosomes in a pH-dependent manner (16). By means of its enzymatic activity, heparanase remodels the endothelial glycocalyx by degradation of heparan sulfate proteoglycans, thereby promoting the release and diffusion of several heparan sulfate linked molecules such as growth factors, cytokines, and enzymes (17). Heparanase plays a crucial role in various physiological and pathological conditions including inflammation, wound healing, tumor angiogenesis, and metastasis (14). Notably, the degradation of heparan sulfate proteoglycans (i.e., syndecan 1) significantly contributes to capillary leakage, platelet aggregation, coagulation, and loss of vascular tone (18, 19) and liberates highly immune-potent circulating heparan sulfate (20). Although understanding the association between heparanase and the clinical course after TAAA repair may help to identify new biomarkers as well as new therapeutic targets, the role of heparanase in the inflammatory response and clinical course after TAAA repair is unknown.

The present study was designed to (i) study perioperative serum levels of heparanase, heparan sulfate, and syndecan-1 in patients undergoing open or endovascular TAAA repair and (ii) evaluate the association between heparanase and the clinical course after TAAA repair. Having discovered that serum levels of heparanase and heparan sulfate are associated with the inflammatory response and clinical course after open and endovascular TAAA repair, we then investigated (in a reverse-translational approach) the systemic inflammatory response and renal injury/dysfunction induced by heparanase and heparan sulfate in mice.

## Materials and Methods

### Use of Human Subjects—Ethic Statement

This study was carried out in accordance with the recommendations of the local ethics committee of University Hospital Aachen with written informed consent from all subjects. All subjects gave written informed consent in accordance with the Declaration of Helsinki. The protocol was approved by the local ethics committee (University Hospital Aachen, EK004/14).

### Use of Experimental Animals—Ethic Statement

This study was carried out in accordance with the recommendations of “Animal Use and Care Committee” in accordance with the derivatives of both the “Home Office guidance on the Operation of Animals (Scientific Procedures) Act 1986” and the “Guide for the Care and Use of Laboratory Animals” of the National Research Council. The protocol was approved by the Animal Welfare Ethics Review Board of Queen Mary University of London, and the study was performed under license issued by home office (Procedure Project License; PPL:70/7348).

### Clinical Study

Between March 2014 and June 2015, we prospectively included 27 consecutive patients undergoing conventional open (*n* = 17, open) and endovascular (*n* = 10, endovascular) TAAA repair. The exclusion criteria were age <18 years, organ transplantation, pregnancy, or receiving palliative care. Serum samples were obtained perioperatively [preoperatively as well as 6 and 72 h after admission on the intensive care unit (ICU)] to determine levels of heparanase, heparan sulfate, and syndecan-1. Laboratory and clinical parameters were extracted from medical records and electronic bedside flow charts [IntelliSpace Critical Care and Anesthesia; Philips Healthcare, Andover, MA, USA]. Definition of acute kidney injury was defined according to the KDIGO clinical practice guidelines for acute kidney injury (serum creatinine >1.5 times baseline and/or urine output <0.5 ml/kg/h for 6–12 h) (21). Lactate clearance was defined as the percent change of lactate level after 6 h compared to a baseline measurement at ICU admission (22). A positive value denotes a decrease or clearance of lactate, whereas a negative value denotes an increase in lactate after 6 h post-ICU admission. All participants had a 90-day follow-up assessment of survival and quality of life using the EuroQoL (EQ)-5D. The EQ-5D includes the EQ-VAS, a visual analog scale ranging current overall health by one single number on a scale from 0 (worst imaginable health state) to 100 (best imaginable health state) [www.euroqol.org; (23)].

### Animal Experiments

This study was carried out on 2-month-old male C57BL/6J mice (Charles River, Kent, UK) weighing 25–30 g, receiving a standard diet and water *ad libitum*. Mice received either heparanase (1 U i.v.; n = 8), heparan sulfate (1 mg i.v., *n* = 8), or their vehicle (0.9% saline, *n* = 8). 24 h later, serum samples were collected for quantification of inflammatory cytokines, levels of heparanase and heparan sulfate, heparanase activity, and renal injury/dysfunction. Cytokine profile measurements were performed as described before (24, 25). Serum levels of heparanase and heparan sulfate were determined using ELISA (AMS Biotechnology, Oxon, UK) according to the manufacturer’s instructions and described previously (26, 27). Heparanase enzymatic activity (heparan sulfate degradation activity) was determined using a commercial available heparanase assay kit (AMS Biotechnology, Oxon, UK) and is expressed as the amount of liberated heparan sulfate (ng) per amount of heparanase (mg). Biochemical markers of renal injury/function (serum urea and serum creatinine) were measured in a blinded fashion by a commercial veterinary testing laboratory (IDEXX Ltd., UK).

### Reagents and Compounds

Reagents and compounds were purchased from Sigma Aldrich (Poole, Dorset, UK), unless otherwise stated.

### Biomarker Assay

The amount of heparanase, heparan sulfate, and syndecan-1 in serum was determined using ELISA (AMS Biotechnology, Oxon, UK) according to the manufacturer’s instructions and described recently (26, 27). Briefly, a total of 100 µL of standards or samples were added to the wells followed by the addition of 100 µL detection reagent A. Serum was diluted for the measurements (heparanase [1:2]; heparan sulfate [1:100]; syndecan-1 [1:50]). The plate was incubated for 1 h at 37°C. After three wash steps with the supplied wash solution, 100 µL detection reagent B was added to each well. The plate was incubated for 30 min at 37°C. 90 µL substrate solution was added to each well before the reaction was halted with stop solution after 10–12 min. The absorbance was measured at 450 nm on a microplate reader (Sunrise Tecan, Crailsheim, Germany).

### Statistical Analyses

Values are expressed as medians and interquartile ranges (IQR), counts and percent, or mean and SEM of *n* observations where appropriated. Group comparisons of continuous variables were performed using the Kruskal–Wallis test Dunn’s multiple comparisons test. The comparisons of categorical variables between groups were performed using Chi-square test. Logistic regression was used to evaluate endothelial markers for the prediction of vasopressor need, and receiver operating characteristic (ROC) curves were constructed for illustration. The area under the ROC curve (AUC, or C index) was given as an effect measurement. All statistical tests were two-tailed, and a two-sided *p*-value equal or below 0.05 was considered significant. The statistical analyses were performed using IBM SPSS Statistics 22.1 (IBM, New York, NY, USA) and GraphPad Prism Version 5.01 (Graphpad Software, San Diego, CA, USA).

## Results

### Study Population

Patients (74% male) were 63 [57–73] (median, IQR) years old, with significant older patients undergoing endovascular compared to open TAAA repair (*p* < 0.01; Table 1). The average body mass index (BMI) was 26.3 [23.7–28.3] kg/m^2^ (*p* = 0.51 between groups). Patients undergoing open repair showed significantly higher levels of serum-lactate on admission to ICU, compared to patients after endovascular repair (3.6 mM/L [1.3–6.1] vs. 0.5 [0.3–0.5]; *p* = 0.03; Table 2).

**Table 1:** Baseline and intraoperative characteristics.

	Total (*n* = 27)	Open thoracoabdominal aortic aneurysm (TAAA)-repair (*n* = 17)	Endovascular TAAA repair (*n* = 10)	*p*-Value
Age (years) (IQR)	63.0 (57.0–73.0)	63.0 (51.5–64.5)	72.5 (66.5–76.3)	0.01
Male (%)	20 (74.1)	13 (76.5)	7 (70.0)	0.71
BMI (kg/m^2^) (IQR)	26.3 (23.7–28.3)	26.3 (22.3–28.5)	27.1 (23.6–29.0)	0.51
Hypertension (%)	26 (96.3)	17 (100.0)	9 (90.0)	0.18
Cerebrovascular disease (%)	6 (22.2)	6 (22.0)	0 (0.0)	0.03
COPD (%)	9 (33.3)	6 (35.3)	3 (30.0)	0.16
Coronary artery disease (%)	10 (37.0)	7 (41.2)	3 (30.0)	0.56
Diabetes (%)	4 (14.8)	2 (11.8)	2 (20.0)	0.56
Current smokers	9 (33.3)	6 (35.3)	3 (30.0)	0.78
Operation time (min) (IQR)	420.0 (360.0–510.0)	435.0 (374.0–525.0)	390.0 (345.0–487.5)	0.65
CPB time (min) (IQR)	75.0 (0.0–116.0)	90.0 (37.5–146.0)	–	–
Aortic clamping time (min) (IQR)	70.0 (0.0–125.0)	70.0 (0.0–117.0)	–	–
Crystalloids (L) (IQR)	3.5 (1.5–4.5)	4.0 (1.5–5.0)	3.5 (2.0–3.9)	0.14
Colloids (L) (IQR)	0.5 (0.0–1.0)	0.5 (0.0–1.0)	0.5 (0.5–1.3)	0.90
Transfusion FFP (units) (IQR)	5.0 (0.0–16.0)	10.0 (0.0–18.0)	2.5 (0.0–5.8)	0.05
Transfusion RBCC (units) (IQR)	1.0 (0.0–6.0)	0.0 (1.0–7.0)	2.5 (0.0–5.8)	0.27
Transfusion PC (units) (IQR)	0.0 (0.0–2.0)	2.5 (0.0–5.8)	2.0 (0.0–2.0)	0.04

*Categorical and continuous variables are presented as n (%) and median [interquartile ranges (IQR)], respectively. Kruskal–Wallis test was used to compare categorical and continuous variables between patients undergoing open or endovascular TAAA repair, respectively*.

*BMI, body mass index; COPD, chronic obstructive pulmonary disease; CPB, cardiopulmonary bypass; FFP, fresh frozen plasma; PC, platelet concentrate; RBCC, red blood cell concentrate*.

**Table 2:** Postoperative characteristics and outcome.

	Total (*n* = 27)	Open thoracoabdominal aortic aneurysm (TAAA)-repair (*n* = 17)	Endovascular TAAA repair (*n* = 10)	*p*-Value
Creatinine (mg/dL) (IQR)	0.9 (0.6–1.7)	1.1 (0.7–1.4)	0.6 (0.3–0.6)	0.14
Urea (mg/dL) (IQR)	30.5 (20.8–37.0)	30.5 (19.8–35.3)	35.5 (29.3–41.0)	0.04
Hemoglobin (g/dL) (IQR)	10.9 (9.9–12.1)	11.2 (10.0–12.4)	10.4 (9.7–11.0)	0.24
Platelets (10^9^ cells/L) (IQR)	125.5 (99.3–138.5)	125.5 (92.3–138.5)	129.0 (106.3–166.8)	0.27
White cells (10^9^ cells/L) (IQR)	8.8 (6.4–12.0)	7.7 (6.1–12.1)	12.0 (6.0–12.0)	0.73
ALT (U/L) (IQR)	22.5 (16.8–46.8)	22.5 (17.0–46.8)	20.5 (9.3–66.3)	0.16
AST (U/L) (IQR)	34.5 (26.8–93.8)	34.5 (26.8–93.8)	51.5 (20.8–97.3)	0.07
Gamma-GT (U/L) (IQR)	16.5 (12.8–29.0)	16.5 (12.0–27.5)	20.5 (13.3–37.5)	0.63
Bilirubin (mg/dL) (IQR)	1.0 (0.4–1.6)	1.2 (0.7–1.6)	0.6 (0.3–2.6)	0.03
Interleukin-6 (pg/mL) (IQR)	111.3 (16.1–184.2)	113.4 (60.2–229.6)	43.4 (4.8–164.0)	0.20
PCT (ng/mL) (IQR)	0.03 (0.03–0.07)	0.03 (0.03–0.18)	0.04 (0.02–0.04)	0.50
CRP (mg/dL) (IQR)	5.1 (3.8–5.6)	5.3 (4.0–19.4)	0.8 (0.4–0.8)	0.77
Lactate (mM/L) (IQR)	1.8 (0.8–6.0)	3.6 (1.3–6.1)	0.5 (0.3–0.5)	0.03
Lactate clearance (%) (IQR)	−25.0 (−55.0–8.3)	−37.5 (−63.4–6.3)	−11.0 (−36.4–32.3)	0.10
Cristalloids within first 24 h (L) (IQR)	2.7 (2.2–3.6)	2.7 (2.2–3.8)	2.8 (2.2–3.2)	0.05
Colloids within first 24 h (L) (IQR)	0.5 (0.0–1.1)	0.8 (0.4–1.5)	0.3 (0.0–0.5)	0.14
Diuresis within first 24 h (L) (IQR)	1.3 (0.7–2.0)	1.3 (0.9–2.6)	1.0 (0.7–1.3)	0.51
Maximal Noradrenaline within first 24 h (μg/kg/min) (IQR)	0.06 (0.01–0.34)	0.08 (0.02–0.29)	0.03 (0.0–0.44)	0.81
SAPS (points) (IQR)	44.0 (21.0–48.0)	44.0 (19.5–47.0)	44.0 (22.5–48.3)	0.51
SOFA (points) (IQR)	8.0 (4.0–11.0)	6.0 (4.5–11.0)	8.5 (2.8–11.3)	0.80
APACHE II (points) (IQR)	21.0 (12.0–25.0)	19.0 (12.5–24.0)	22.0 (11.8–28.0)	0.45
MV (hours) (IQR)	16.8 (11.0–42.3)	22.8 (12.4–544.8)	15.4 (9.7–26.5)	0.36
Acute kidney injury (%)	5 (18.5)	3 (17.6)	2 (20.0)	0.69
LOS intensive care unit (days) (IQR)	4.0 (1.0–7.0)	4.0 (1.5–6.5)	3.5 (1.0–11.5)	0.82
90-day EQ-VAS score (points) (IQR)	55.0 (50.0–72.5)	65.0 (50.0–85.0)	50.0 (13.8–61.3)	0.06
90-day mortality (%)	3 (11.1)	3 (17.6)	0 (0.0)	0.13

*Categorical and continuous variables are presented as n (%) and median (interquartile ranges, IQR), respectively. Kruskal–Wallis test was used to compare categorical and continuous variables between patients undergoing open or endovascular TAAA repair, respectively*.

*ALT, alanine aminotransferase; AST, aspartate aminotransferase; APACHE II, Acute Physiology and Chronic Health Evaluation II Score; BMI, body mass index; CRP, C-reactive protein; eGFR, estimated glomerular filtration rates; EQ-VAS, EuroQol visual analog scale; gamma-GT, gamma-glutamyl transferase; IL, interleukin; LOS, length of stay; MV, mechanical ventilation; PCT, procalcitonin; SAPS, Simplified Acute Physiology Score; SOFA, Sequential Organ Failure Assessment Score*.

### Clinical Course and Outcome

According to the KDIGO clinical practice guidelines for acute kidney injury, 18.5% (5/27) of the patients developed postoperative acute kidney injury, with no differences detected between patients undergoing open or endovascular TAAA repair (*p* > 0.05; Table 2). The length of stay (LOS) in the ICU, the Simplified Acute Physiology Score (SAPS), the Sequential Organ Failure Assessment Score (SOFA), and the Acute Physiology and Chronic Health Evaluation II Score (APACHE II) on the day of admission to the ICU did also not differ between the groups (all *p* > 0.05; Table 2). Moreover, the 90-day EQ-VAS score as well as the 90-day mortality rate did not significantly differ between the groups (all *p* > 0.05; Table 2).

### Perioperative Serum Levels of Heparanase, Heparan Sulfate, and Syndecan-1

**Figure 1:**
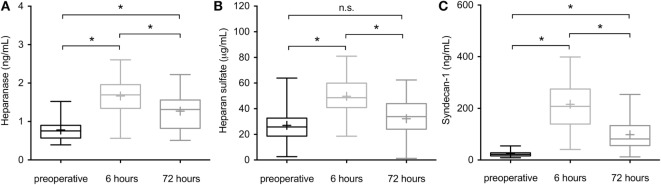
Perioperative serum levels of heparanase, heparan sulfate, and syndecan-1 in patients undergoing open or endovascular thoracoabdominal aortic aneurysm (TAAA)-repair. Serum levels of **(A)** heparanase, **(B)** heparan sulfate, and **(C)** syndecan-1 were assessed in patients undergoing open or endovascular TAAA repair (*n* = 27) preoperatively, 6 and 72 h after intensive care unit admission. Data are expressed as Box and Whisker min to max for *n* number of observations. + indicates the median. *p < 0.05; n.s., non-significant (Kruskal–Wallis test with Dunn’s multiple comparisons test).

Linear regression analysis including age, gender, and BMI revealed no significant influence on the investigated endothelial markers (all *p* > 0.05). Serum levels of heparanase had significantly increased 6 h after admission to the ICU when compared to preoperative values (1.4 ng/mL [1.1–2.0] vs. 0.7 [0.5–0.9], *p* < 0.0001) and returned to normal values by 72 h after ICU admission, compared to 6 h after ICU admission (0.8 ng/mL [0.5–1.1] vs. 0.7 [0.5–0.9], *p* = 0.002; Figure 1A). Similarly, serum levels of heparan sulfate significantly increased at 6 h compared to preoperative values (54.6 µg/mL [40.6–67.6] vs. 23.9 [21.4–49.0], *p* < 0.0001) and returned to normal within 72 h after ICU admission (38.7 µg/mL [24.7–46.0], *p* = 0.002; Figure 1B). At 72 h after admission to the ICU, serum levels of heparan sulfate did not differ significantly from the preoperative values (*p* = 0.69; Figure 1B). Comparable to heparanase and heparan sulfate, serum levels of syndecan-1 significantly increased 6 h after admission to the ICU when compared to preoperative values (244.7 ng/mL [125.1–277.4] vs. 23.0 [16.7–32.4], *p* < 0.0001) and returned to normal within 72 h when compared to the 6 h value (66.3 ng/mL [30.2–112.3] vs. 23.0 [16.7–32.4], *p* = 0.0002; Figure 1C). However, serum concentrations of heparanase, heparan sulfate, as well as syndecan-1 did not differ significantly between the groups throughout the study period (all *p* > 0.05; Table 3).

**Table 3:** Serum concentrations of endothelial markers.

	Total (*n* = 27)	Open thoracoabdominal aortic aneurysm (TAAA)-repair (*n* = 17)	Endovascular TAAA repair (*n* = 10)	*p*-Value
Heparanase preoperative (ng/mL) (IQR)	0.7 (0.5–0.9)	0.9 (0.7–1.0)	0.6 (0.5–0.7)	0.69
Heparanase 6 h after ICU admission (ng/mL) (IQR)	1.4 (1.1–2.0)	1.6 (1.4–2.0)	1.1 (0.8–1.6)	0.73
Heparanase 72 h after ICU admission (ng/mL) (IQR)	0.8 (0.5–1.1)	1.0 (0.9–1.6)	0.6 (0.5–0.8)	0.43
Heparan sulfate preoperative (μg/mL) (IQR)	23.9 (21.4–49.0)	36.7 (23.9–50.0)	22.4 (14.2–43.3)	0.47
Heparan sulfate 6 h after ICU admission (μg/mL) (IQR)	54.6 (40.6–67.6)	57.8 (44.5–73.4)	51.3 (31.9–66.5)	0.13
Heparan sulfate 72 h after ICU admission (μg/mL) (IQR)	38.7 (24.7–46.0)	31.8 (24.5–42.1)	42.9 (24.6–48.8)	0.43
Syndecan-1 preoperative (ng/mL) (IQR)	23.0 (16.7–32.4)	21.9 (16.5–41.7)	24.1 (15.3–34.4)	0.13
Syndecan-1 6 h after ICU admission (ng/mL) (IQR)	244.7 (125.1–277.4)	138.6 (62.7–270.5)	260.9 (218.1–304.6)	0.34
Syndecan-1 72 h after ICU admission (ng/mL) (IQR)	66.3 (30.2–112.3)	31.0 (20.1–119.2)	72.7 (48.9–131.7)	0.69

*Variables are presented as median and interquartile ranges (IQR). Kruskal–Wallis test was used to compare variables between patients undergoing open or endovascular TAAA repair, respectively*.

*ICU, intensive care unit*.

### Significance of Perioperative Heparanase Levels on Postoperative Clinical Course

**Figure 2:**
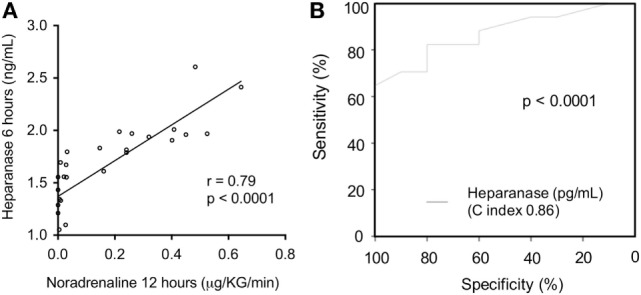
Association between postoperative serum levels of heparanase and the need of vasopressor. Serum levels of heparanase were assessed in patients undergoing open or endovascular thoracoabdominal aortic aneurysm repair (*n* = 27) 6 h after intensive care unit (ICU)-admission. **(A)** Correlation of serum levels of heparanase 6 h post-ICU admission with the noradrenaline dose 12 h after ICU admission. **(B)** Receiver operating characteristic curve for the value of heparanase 6 h after ICU admission in predicting the need of vasopressor within the first 24 h after ICU admission.

6 h after ICU admission heparanase serum levels showed a strong correlation with the dose of noradrenalin 12 h after admission (Figure 2A). Furthermore, heparanase serum levels at 6 h post-ICU admission were a better predictor of vasopressor need within the first 24 h (Figure 2B) than serum lactate (AUC 0.86, *p* < 0.0001 vs. AUC 0.40, *p* = 0.46, respectively) or interleukin (IL)-6 (AUC 0.48, *p* = 0.89).

### Significance of Perioperative Heparan Sulfate Levels on Postoperative Clinical Course

**Figure 3:**
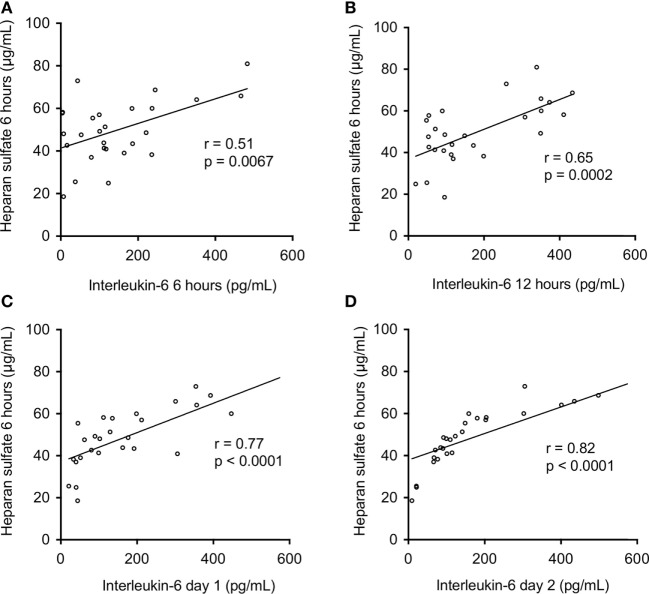
Association between postoperative serum levels of heparan sulfate and interleukine-6. Serum levels of heparan sulfate were assessed in patients undergoing open or endovascular thoracoabdominal aortic aneurysm repair (*n* = 27) 6 h after intensive care unit (ICU) admission. Correlation between serum levels of heparan sulfate 6 h after ICU admission and interleukin-6 at **(A)** 6 h, **(B)** 12 h, **(C)** day 1, and **(D)** day 2 after ICU admission.

**Figure 4:**
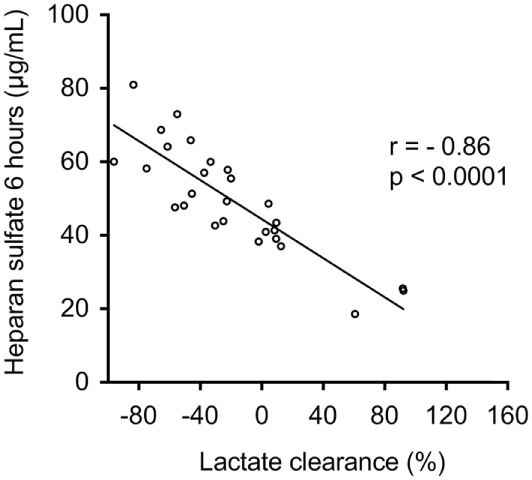
Association between serum levels of heparan sulfate and lactate clearance. Serum levels of heparan sulfate were assessed in patients undergoing open or endovascular thoracoabdominal aortic aneurysm repair (*n* = 27) 6 h after intensive care unit (ICU) admission. Correlation of serum levels of heparan sulfate 6 h after ICU admission with lactate clearance. Lactate clearance was defined as lactate levels at the admission to the ICU divided by lactate at hour 6 after ICU admission multiplied by 100 minus 100 (22).

Figure 3 shows the correlation between heparan sulfate serum levels and IL-6 levels on day 0, 1, and 2 after ICU admission. Heparan sulfate serum levels 6 h after ICU admission showed a strong correlation with IL-6 levels 6 h and 12 h after ICU admission (*r* = 0.51, *p* = 0.0067 and *r* = 0.65, *p* = 0.0002, respectively) as well as on day 1 and 2 after ICU admission (*r* = 0.77, *p* < 0.0001 and *r* = 0.82, *p* > 0.0001, respectively). Furthermore, heparan sulfate serum levels 6 h after ICU admission showed a strong negative correlation with lactate clearance during the first 6 h post ICU admission (*r* = −0.86, *p* < 0.0001; Figure 4).

### Effects of Heparanase and Heparan Sulfate on Serum Heparanase Activity and Serum Heparan Sulfate Levels in Mice

**Figure 5:**
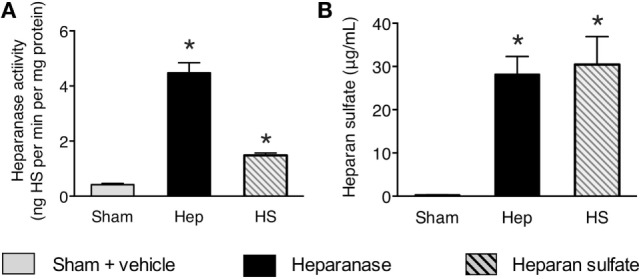
Effects of heparanase or heparan sulfate on serum heparanase activity and serum heparan sulfate levels in C57BL/6J mice. **(A)** Heparan sulfate degradation activity and **(B)** heparan sulfate levels were assessed 24 h subsequent to either vehicle (sham), heparanase (1 U i.v.; Hep), or heparan sulfate (1 mg i.v.; HS) administration in 2-month-old male C57BL/6J mice. The following groups were studied: C57BL/6J vehicle (*n* = 8); C57BL/6J heparanase (*n* = 8); C57BL/6J heparan sulfate (*n* = 8). Data are expressed as means ± SEM for *n* number of observations. *p < 0.05 vs. C57BL/6J vehicle (Kruskal–Wallis test with Dunn’s multiple comparisons test).

Having discovered that the serum levels of heparanase and heparan sulfate are associated with the inflammatory response and clinical course after open and endovascular TAAA repair, we next investigated (in a reverse-translational approach) the effects of the administration of heparanase or heparan sulfate on serum heparanase activity and serum heparan sulfate levels in mice. When compared with sham-animals (vehicle administration), mice subjected to heparanase (1 U; i.v.) or heparan sulfate (1 mg; i.v.), showed within 24 h a significant “activation” of heparanase indicated by a significantly higher enzymatic serum heparanase degradation activity (Figure 5). Moreover, when compared with sham-animals, mice subjected to heparanase or heparan sulfate, at 24 h showed significantly higher serum levels of heparan sulfate (Figure 5).

### Effects of Heparanase and Heparan Sulfate on Systemic Inflammatory Response in Mice

**Figure 6:**
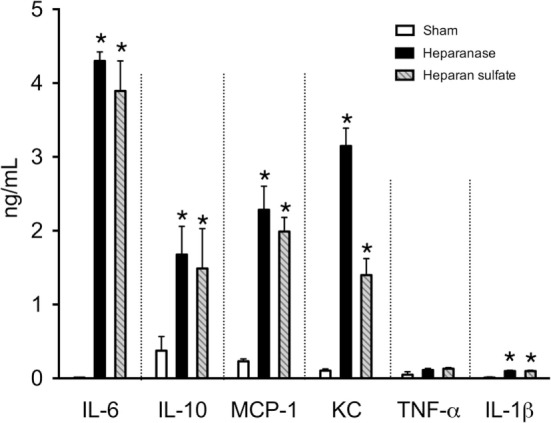
Effects of heparanase or heparan sulfate on systemic inflammatory response in C57BL/6J mice. Serum levels of interleukine-6 (IL-6), IL-10, monocyte chemoattractant protein-1 (MCP-1), C-X-C motif ligand 1, tumor necrosis factor-α (TNF-α), and IL-1β were assessed 24 h subsequent to either vehicle (sham), heparanase (1 U i.v.; Hep), or heparan sulfate (1 mg i.v.; HS) administration in 2-month-old male C57BL/6J mice. The following groups were studied: C57BL/6J vehicle (*n* = 8); C57BL/6J heparanase (*n* = 8); C57BL/6J heparan sulfate (*n* = 8). Data are expressed as means ± SEM for *n* number of observations. *p < 0.05 vs. C57BL/6J vehicle (Kruskal–Wallis test with Dunn’s multiple comparisons test).

Next, we investigated the effects of heparanase and heparan sulfate on systemic inflammatory response in mice, indicated by serum levels of pro- and anti-inflammatory cytokines in mice. When compared with sham-animals, mice subjected to heparanase or heparan sulfate demonstrated a significant increase in IL-6 (*p* < 0.0001), IL-10 (*p* < 0.05), monocyte chemoattractant protein-1 (*p* < 0.0001), C-X-C motif ligand 1 (*p* < 0.0001), and IL-1β (*p* < 0.0001), indicating a strong, systemic pro-inflammatory response (Figure 6). There were no significant differences in the levels of tumor necrosis factor-α (*p* = 0.18) (Figure 6).

### Effects of Heparanase and Heparan Sulfate on Renal Injury/Dysfunction in Mice

**Figure 7:**
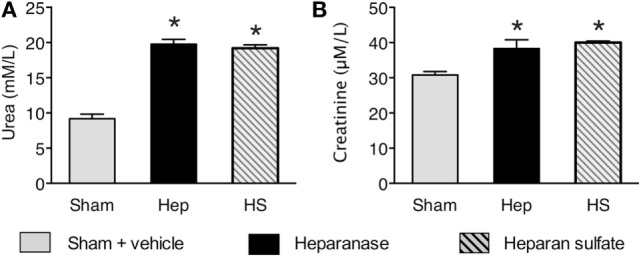
Effects of heparanase and heparan sulfate on renal injury/dysfunction in C57BL/6J mice. Serum levels of **(A)** urea and **(B)** creatinine were assessed 24 h subsequent to either vehicle (sham), heparanase (1 U i.v.; Hep), or heparan sulfate (1 mg i.v.; HS) administration in 2-month-old male C57BL/6J mice. The following groups were studied: C57BL/6J vehicle (*n* = 8); C57BL/6J heparanase (*n* = 8); C57BL/6J heparan sulfate (*n* = 8). Data are expressed as means ± SEM for *n* number of observations. *p < 0.05 vs. C57BL/6J vehicle (Kruskal–Wallis test with Dunn’s multiple comparisons test).

Having discovered that heparanase and heparan sulfate induce a pro-inflammatory response *in vivo*, we next questioned whether heparanase and heparan sulfate induce a renal injury/dysfunction in mice. When compared with sham animals, mice subjected to heparanase or heparan sulfate, at 24 h, developed significant renal dysfunction, indicated by a significant increase in serum urea and serum creatinine (Figure 7, *p* < 0.0001).

## Discussion

We report here for the first time that serum levels of heparanase, heparan sulfate, and syndecan-1 are positively correlated with (and to some degree predict) both systemic inflammatory response and clinical outcome after open and endovascular TAAA repair. Most notably, postoperative heparanase was highly predictive for postoperative vasopressor requirements and levels of heparan sulfate showed a strong negative correlation with lactate clearance after ICU admission. Using a reverse-translational approach, we further identified heparanase and heparan sulfate as relevant endogenous “danger molecules,” which are sufficient (in the absence of any other pro-inflammatory molecules) to induce a systemic inflammatory response and a small degree of renal dysfunction in mice.

Activated platelets and endothelial cells are the most prominent sources of heparanase, which is the only known mammalian endoglycosidase that plays a role in the shedding of the endothelial glycocalyx (13). Although elevated levels of the glycocalyx have been reported in different clinical settings with injury, such as sepsis or major abdominal surgery (28), our data indicate for the first time a crucial role of heparanase in both systemic inflammation and morbidity after both open and endovascular TAAA repair (Figures 1–3). Notably, we detected no differences in the serum levels of heparanase between patients undergoing open or endovascular TAAA repair throughout the study period (Table 3), raising the question about the (different) mechanisms that contribute to the release of heparanase in open and endovascular procedures. As a result of its unique position between the blood and the vessel wall, the endothelial glycocalyx plays a pivotal role during injury, both as target and propagator of the systemic inflammatory response (12). One of the most prominent characteristics of open TAAA repair is the phenomenon of I/R-injury that happens either at the time of clamping and following the removal of the aortic clamp or during CPB with consecutive activation of platelets (29). In this regard, Rehm and colleagues investigated heparan sulfate levels in arterial blood of patients undergoing surgery of the ascending aorta. During early reperfusion after global ischemia with circulatory arrest, levels of heparan sulfate showed a 10-fold increase, compared to baseline levels (30). Furthermore, electron microscopy in guinea pigs revealed a shedding of the glycocalyx with a consecutive release of heparan sulfate caused by I/R-injury (30). Thus, it is likely that the observed release of heparanase during conventional open TAAA repair is due to the activation of platelets induced by I/R-injury and extracorporeal circulation (31, 32). Furthermore, the connection of platelets to the new vascular surface (within the graft material) is a conceivable reason for the detected effects; however, further studies are warranted to clarify this. In contrast to these indirect mechanisms in open TAAA repair, the discovered release of heparanase in patients undergoing endovascular TAAA repair may be explained by (direct) “activation” of the endothelium with consecutive release of heparanase by endothelial cells. In this setting, the most important “activators” of the endothelium are the contrast media, the endothelial manipulation with the introducer sheaths, the inner surface of the endograft material *per se*, the manipulation of the mural thrombus with the consecutive release of thrombotic contents, and the radial forces in the landing zones during stent implantation (9). Thus, it is unclear whether the inner surface of the graft material (which exists in both types of TAAA repair in a similar extend) or totally different mechanisms is responsible for the release of heparanase in open and endovascular TAAA repair.

Postoperative hypotension, which requires vasopressor support, is a frequent complication after open and endovascular TAAA repair and an independent risk factor for injury/dysfunction of lung, liver, and kidney (4). Vascular leakages with edema formation as well as the excessive production of endothelial nitric oxide (NO), probably by the inducible isoform of NO synthase (33), are the key pathophysiological drivers of vascular decompensation secondary to an excessive, postoperative systemic inflammatory response (34). Heparanase is a strong inducer of vascular hyperpermeability and regulates the production of NO due to the enzymatic degradation of glycosaminoglycans in the vascular subendothelial basement membrane, suggesting that serum levels of heparanase may be associated with postoperative hypotension in patients undergoing TAAA repair. Indeed, we found a strong correlation of heparanase levels with the dose of noradrenaline required to maintain blood pressure at 12 h after ICU admission (Figure 2A). Moreover, we found that postoperative heparanase levels predict the vasopressor requirements throughout the first 24 h of ICU stay (Figure 2B). As postoperative hypoperfusion, due to macro- and/or microcirculatory dysfunction and hypotension, results in elevated lactate levels and contribute to multiple organ failure after major surgery (35), we further evaluated lactate clearance as a surrogate marker for the magnitude and duration of global tissue hypoxia (22, 36). Here, we show that levels of heparan sulfate, released by heparanase, showed a strong negative correlation with lactate clearance (Figure 4). Thus, our results suggest that postoperative hypotension/hypoperfusion after TAAA repair is (at least in part) due to the release of heparanase and notably to the consecutive release of heparan sulfate.

Multiple factors have been proposed to cause the systemic inflammatory response after open and endovascular TAAA repair (9). The roles of heparan sulfate, liberated by heparanase, however, are unknown. Here, we found a strong correlation of heparan sulfate levels with IL-6 levels on day 0, 1, and 2 after the admission to the ICU (Figure 3), suggesting that circulating heparan sulfate may play a pivotal role in the induction of postoperative systemic inflammation in patients undergoing TAAA repair. To support this hypothesis (and to gain a better insight into the roles of heparan and heparan sulfate in systemic inflammation), we have used a reverse translational approach to investigate the effects of the systemic administration of either heparanase or heparan sulfate on systemic inflammatory response and renal injury/dysfunction in mice. We first evaluated whether heparanase administration may, in principle, be able to release heparan sulfate from endogenous stores of heparan sulfate (cell-bound heparan sulfate proteoglycans). The intravenous injection of heparanase resulted in significantly elevated serum levels of heparan sulfate that were quantitatively similar to the serum levels of heparan sulfate in mice subjected to heparan sulfate (Figure 5B). This result indicates that the administration of heparanase can, in principle, release relatively large amounts of heparan sulfate *in vivo*. Interestingly, the administration of heparan sulfate, in turn, significantly increased heparanase degradation activity (Figure 5A). As pro-inflammatory cytokines (i.e., IL-6) induce the cleavage of the 65-kDa heparanase to its active 50-kDa form (11), this effect may be explained by the inflammatory response caused by the administration of heparan sulfate. Indeed, we detected a strong inflammatory response in mice after administration of either heparanase or heparan sulfate (Figure 6). These *in vivo* data extend earlier *in vitro* work showing that soluble heparan sulfate generated by heparanase trigger the release of pro-inflammatory cytokines in immune cells (20). In addition, our group recently reported that heparan sulfate in serum of septic shock patients induce an inflammatory response in cultured cardiomyocytes, as shown by a significant increase in IL-6. Eliminating circulating heparan sulfate from serum of septic patients, however, significantly attenuates the inflammatory response (26).

Having discovered that heparanase and heparan sulfate induce a pro-inflammatory response *in vivo*, we next questioned whether heparanase and/or heparan sulfate induce renal injury/dysfunction in mice, which is a common postoperative complication in patients undergoing open or endovascular TAAA repair (4). In mice treated with either heparanase or heparan sulfate, we found (24 h after challenge) a small, but significant (and quantitatively similar) increase in both serum urea and creatinine, indicating the development of a small, but significant degree of renal dysfunction (Figure 7). Lygizoz et al. recently showed that glomerular heparanase is activated during septic shock and associated with the loss of glomerular filtration (37). Although the underlying mechanism to the observed heparanase-related renal dysfunction remain unclear, it is likely that the degradation of glomerular glycocalyx as well as a heparanase-mediated alteration of arteriole tone (*via* altered NO production) may represent potential mechanisms underlying heparanase-associated renal dysfunction (38). Further translational studies should pursue these and other potential mechanisms underlying heparanase-related renal dysfunction to gain a better mechanistic understanding of the role of heparanase and heparan sulfate in multiple organ injury/dysfunction.

In conclusion, although limited by a small sample size due to the rare procedures investigated, our results show that heparanase and heparan sulfate exhibit a substantial role as clinically relevant danger molecules and may serve as both, promising biomarkers and therapeutic targets in patients undergoing open and endovascular TAAA repair. Currently, there is no specific treatment for postoperative organ failure/dysfunction after TAAA repair, which is associated with high morbidity and mortality. We recently reported that a synthetic host-defense peptide that targets heparanase and heparan sulfate attenuates the organ injury/dysfunction associated with hemorrhagic shock (39, 40). Thus, we postulate that synthetic host-defense peptides may be promising therapeutics against postoperative organ failure/dysfunction after TAAA repair.

## Ethics Statement

*Use of human subjects*—*ethic statement*: This study was carried out in accordance with the recommendations of the local ethics committee of University Hospital Aachen with written informed consent from all subjects. All subjects gave written informed consent in accordance with the Declaration of Helsinki. The protocol was approved by the local ethics committee (University Hospital Aachen, EK004/14). *Use of experimental animals—ethic statement*: This study was carried out in accordance with the recommendations of “Animal Use and Care Committee” in accordance with the derivatives of both, the “Home Office guidance on the Operation of Animals (Scientific Procedures) Act 1986,” and the “Guide for the Care and Use of Laboratory Animals” of the National Research Council. The protocol was approved by the Animal Welfare Ethics Review Board of Queen Mary University of London and the study was performed under license issued by home office (Procedure Project License; PPL:70/7348).

## Author Contributions

Conception and design: LM, CT, and TS. Clinical study and patient data analyses: LM, AG, JL, JV, JK, GM, and MJ. Animal experiments: LM and JC. Drafting and/or revising the manuscript for important intellectual content: LM, CT, and TS. All authors reviewed and finally approved the manuscript.

## Conflict of Interest Statement

The authors declare that the research was conducted in the absence of any commercial or financial relationships that could be construed as a potential conflict of interest.
